# Short Notices of Hospital Therapeutics

**Published:** 1855-09

**Authors:** 


### SHORT NOTICES OF HOSPITAL THERAPEUTICS.

*Artificial Eyes.*—The following scraps of information as to the employment of artificial eyes, which we have acquired in watching the practice at the Royal Ophthalmic Hospital, may be welcome to some of our readers. (*a*) The success in the deception as to appearance is generally most complete. Several very pleasing cases have fallen under our notice, in which a glass eye, by hiding a loathsome deformity, and restoring personal appearance, became the means of effecting a complete revolution in the worldly prospects of the wearer. (*b*) In order to complete success, it is very desirable that the substitute eye should move well. This, however, is not essential, as should the two eyes not move equally the only defect suggested to the casual observer is that of a slight squint. (*c*) To secure the movements of the artificial organ, the natural globe, in its collapsed state, should, if possible, be retained in order to serve as a stump. This stump or cushion, receives the attached muscles and obeys their movements, of course carrying with the it concave glass eye which has been fitted upon it. (*d*) If the entire globe be diseased, and its removal necessary, the operation should be conducted on the modern plan, viz., by division of the muscles close to their attachments, nothing whatever excepting the globe itself being taken away. By this precaution the muscles will be left in their full length, and, becoming connected, in the course of healing, with the mass of cellular tissue, fat, etc., which remains in the orbit, will constitute a cushion possessed of a certain degree of mobility. (*e*) Glass eyes will not wear for ever. Even with careful patients the artificial eye generally requires to be renewed, or, at least, re-enamelled, once a year. It becomes coated at the back by concretions from the tears, and is then so irritating that its disuse becomes necessary. To obviate this inconvenience, patients should always remove them at night, and have them carefully washed; they should also, if convenient, lay them aside for a few days whenever the eye becomes irritable, or a greater tendency to deposit is observed than usual. (*f*) Among the poor this liability to soon become unwearable is a serious objection to their use. Some Surgeons have, indeed, almost ceased to recommend them to their Hospital patients on this account, reserving their employment for cases in which the sufferer appears more than usually intelligent, and likely to succeed in the management. (*g*) Mr. Gray, (of Goswell street,) the maker of artificial eyes to the Ophthalmic Hospital, informed us, in answer to inquiries on this head, that he thought an artificial eye might, with ordinary care, be kept in a good state at a cost of about fifteen shillings a year. This estimate, of course, applies only to a pauper patient, to whom cost price only would be charged.

*Browning of the conjunctiva from Nitrate of Silver.*—When solutions of nitrate of silver are long employed as eye-drops it is not unusual for a conjunctiva to assume a brown or olive hue. The staining is permanent unless removed by remedies. Mr. Critchett ordered, in a case of this kind which presented itself at the Moorfields Ophthalmic Hospital a few weeks ago, a solution of iodide of potassium (gr. viij. ad ℥j.) to be freely used. He stated that he had seen this lotion diminish the stain, but had never known it effect a complete removal. Cases in which nitrate of silver drops are used should always be carefully watched, in order at once to desist, should this change of color be caused. The sulcus of junction of the ocular and palpebral conjunctiva is the part where the staining commences, and if the Surgeon be particular to inspect this frequently, he will run no risk.

*Mechanical support in case of Pendulous Abdomen.*—There are certain cases of enlarged and pendulous abdomen which, when they occur in women, often excite suspicions either of pregnancy or of ovarian disease. Their true pathological nature we once heard pointedly indicated by a Borough obstetrician in three words—"fat, fæces, flatus." Every one of experience will recognize the condition we refer to, the subjects of which are usually stout phlegmatic women, liable to flatulence and intestinal torpidity. The state is one often found difficult of relief. We observe that Dr. West, at St. Bartholomew's Hospital, insists strongly upon the benefits derivable from affording to the bowels mechanical support. By the use of an abdominal bandage, applied as tightly as it can well be borne, the symptoms often disappear very quickly, and a patient whom nothing could convince that she was not either pregnant or dropsical, may have at once her hopes dissipated and her fears dispelled. The use of tonics, and mild purgatives (*e. g.* aloes) at the same time of course advisable. Dr. West often employs frictions with tincture of aloes over the abdomen, with the object of restoring tone to the muscles of the abdominal wall and the intestines. We believe, however, that he considers mechanical support as the most important part of the plan. —*Medical Times and Gazette.*

